# Catechin and caffeine contents in green tea at different harvest periods and their metabolism in miniature swine

**DOI:** 10.1002/fsn3.1143

**Published:** 2019-07-28

**Authors:** Misato Wakamatsu, Hiroki Yamanouchi, Hisashi Sahara, Takehiro Iwanaga, Rei Kuroda, Ayaka Yamamoto, Yuji Minami, Mitsuhiro Sekijima, Kazuhiko Yamada, Katsuko Kajiya

**Affiliations:** ^1^ Biochemical Science & Technology, Graduate School of Agriculture Kagoshima University Kagoshima Japan; ^2^ Division of Organ Replacement and Xenotransplantation Surgery, Center for Advanced Biomedical Science and Swine Research Kagoshima University Kagoshima Japan; ^3^ Department of Food Science & Biotechnology, Faculty of Agriculture Kagoshima University Kagoshima Japan

**Keywords:** caffeine, catechin, green tea, harvest, miniature swine

## Abstract

The catechin content in green tea leaves varies according to cultivation conditions such as intensity of solar radiation, temperature, and precipitation, and thus, there is ambiguity about the best harvest time for obtaining optimal functional effects. In this study, the Yabukita (ordinary) and Benifuki varieties, which contain methylated catechin, were used to determine the difference in green tea catechins according to harvest times and tea manufacturing processes. Caffeine determination was also carried out to provide information about green tea intake for all age‐groups of children and pregnant women. Determining the quantity of each catechin was difficult because of degradation, polymerization, and isomerization that had occurred during heat‐drying in the refining process. In addition, the absorption of catechin compounds was tested using miniature swine because of their functional and physiological similarity to humans. Benifuki tea leaves contained epigallocatechin‐3‐(3”‐O‐methyl) gallate (EGCg3”Me) instead of epigallocatechin‐3‐(4”‐O‐methyl) gallate (EGCg4”Me). However, EGCg4”Me was detected during the entire intake period, but EGCg3”Me was not detected in the blood of miniature swine fed Benifuki tea. It is possible that the position of the methyl group was modified by the pig metabolism. Furthermore, caffeine from both Yabukita and Benifuki tea varieties was found to be easily accumulated in miniature swine. These results suggest that nonrefined September–October picking tea (autumn and winter tea) of the Benifuki variety is preferable over the Yabukita variety for consumption by children and pregnant women owing to its lower caffeine content and higher content of methylated catechin.

## 1 INTRODUCTION

Tea varieties such as green tea, black tea, and oolong tea are consumed globally, and green tea in particular is consumed in large quantities in Japan. Biofunctional properties of green tea, such as antioxidant effects (Lambert & Yang, 2003; Yokozawa, Cho, Hara, & Kitani, 2000), antibacterial action (Amarowicz, Pegg, & Bautista, 2000; Fukai, Ishigami, & Hara, 1991; Kajiya et al., 2004), hypotensive effects (Yokozawa, Okura, Sakanaka, Ishigaki, & Kim, 1994), blood cholesterol‐reducing effects (Muramatsu, Fukuyo, & Hara, 1986; Murase, Nagasawa, Suzuki, Hase, & Tokimitsu, 2002), and neuroprotective effects (Jain, Siddiqi, & Weisburger, 2006), have been reported, and many of these activities are dependent on catechins, particularly epigallocatechin gallate (EGCg). Furthermore, methylated catechin resulting from partial methylation of the gallate group of EGCg is highly effective against chronic atopic eczema or acute pollinosis (Murase et al., 2002). However, the catechin content in green tea leaves varies according to cultivation conditions such as intensity of solar radiation, temperature, and precipitation, and thus, there is ambiguity about the best harvest time for obtaining optimal functional effects. First picking green tea harvested in April–May has the maximum amount of taste components such as amino acids (Jain et al., 2006; Kuroda & Hara, 2004) and is sold at the highest price. Tea harvested in June is second picking tea, in July–August third picking tea, and in September–October final picking tea (autumn and winter tea). Refinements in tea manufacturing processes also influence the functional effects of green tea. Nonrefined tea contains leaves, stem, and buds, and its shape is inconsistent. On the other hand, refined tea is obtained after sieving, shaping, and drying by heating. Therefore, the Yabukita (ordinary) and Benifuki (methylated catechin (Maeda‐Yamamoto et al., 2009)) varieties were used in this study to investigate how green tea catechins are influenced by harvest times and tea manufacturing processes. The Benifuki tea tested was derived from the Assam variety Benihomare and the Indian Darjeeling variety Makura cd86. Characteristically, the Assam series is deep‐ruby‐colored with a steady, rather bitter flavor, but it has a rich original taste.

Caffeine has been reported to have analgesic, antipyretic (Dascombe & Milton, 1972), and diuretic (Stookey, 1999) effects. It has also been reported to have an awakening effect (LaJambe, Kamimori, Belenky, & Balkin, 2005), with a long‐term risk of high blood pressure (Rodríguez‐Artalejo & Lopez‐Garcia, 2017) and inhibitory effects on fetal development in pregnancy. Therefore, we also investigated the effects of caffeine after intake of green tea, as it may affect all age‐groups, including children and pregnant women.

In Japan, the catechins within green tea leaves have attracted attention. Japanese people consume tea leaves directly; for example, they drink water with dissolved tea leaf powder and sprinkle the powder on ice cream. Catechins, especially methylated catechins, which are found in Benifuki, modulate the allergic response and are implicated in hay fever. Thus, it is important to determine whether the body can properly absorb the components of green tea. However, it is difficult to assess the corresponding metabolism. In the present study, miniature (not micro) swine were used as a model to investigate the metabolism of catechins and caffeine. In contrast to rats and mice often used in metabolic studies, these animals have a cardiovascular system similar to that in humans (functional resemblance). In addition, unlike monkeys, which feed on fruits and nuts, miniature swine are omnivorous like humans and have similar digestive and circulatory systems (physiological resemblance). Because of the ease of rearing management and genetic control, they are widely used in medicine, biology, immunology, pharmacology, and regenerative medicine and can be used as large laboratory animals with a relatively low cost. The only difference between miniature swine and commonly known edible pigs is size, but their biological features such as sex cycle and life span are almost the same.

## 2 MATERIALS AND METHODS

### 2.1 Materials

Catechin ((+)‐C), catechin gallate (Cg), gallocatechin (GC), gallocatechin gallate (GCg), epicatechin (EC), epicatechin gallate (ECg), epigallocatechin (EGC), EGCg, EGC‐3‐(3”‐O‐methyl) gallate (EGCg3”Me), and EGC‐3‐(4”‐O‐methyl) gallate (EGCg4”Me) were purchased from Funakoshi. Caffeine and methanol were purchased from Wako Pure Chemical Industries, and acetonitrile was purchased from Sigma. All other reagents were of special or HPLC grade. Green tea varieties Yabukita and Benifuki harvested at the same time were provided by Kagoshima Seicha.

### 2.2 Tea sample preparation

Nonrefined and refined forms of first, second, third, and final picking tea were used for analysis. Tea infusion samples were prepared by pulverizing 10 mg of green tea in a mill and extracting for 10 min each with 10 ml of warm water at 60, 70, and 80°C. Tea extract samples were prepared by accurately weighing 10 mg of green tea in a conical flask, adding 2 ml of a 1:1 mixture of acetonitrile and water, incubating for 60 min at room temperature (20–25°C) in the dark, and then increasing the volume to 10 ml with distilled water. The thoroughly mixed supernatant solution was used in the experiments.

### 2.3 High‐performance liquid chromatography (HPLC)

After filtration with a 0.45‐μm filter, tea infusion and tea leaf extract samples were analyzed using HPLC (Extrema, Jasco). HPLC analysis conditions were as follows: C18 reverse‐phase column (TSKgel ODS‐100Z, 5 μm, 4.6 mm I.D. × 150 mm; Tosoh) and guard column (TSKgel Guardgel ODS‐100Z, 5 μm, 3.2 mm I.D. × 15 mm; Tosoh) were maintained at 30°C, and detection was performed at 280 nm. The mobile phase was 0.05% phosphoric acid (A) and methanol:acetonitrile (3:2) binary solution system (B) with the following conditions: 0–8 min with B solution 25% constant and a gradient of 8–18 min with a direct increase in B solution up to 75%. Individual compounds were identified with highly selective spectral data in combination with their retention times and an ultraviolet photodiode array detector (UV‐4075 and MD4010, Jasco).

### 2.4 Administration of tea leaves to miniature swine

Male CLAWN miniature swine at 4 months of age and weighing 14 kg were obtained from Kagoshima Miniature Swine Research Center . We used four pigs in each experimental group (Yabukita and Benifuki) and repeated (two‐dose) studies to assess the absorption of tea catechins and caffeine in miniature swine. During the experimental period, body weight (BW), stool condition, and food intake of these animals were observed daily. For longitudinal assessment of samples, an indwelling central venous catheter was placed into the external jugular vein of each animal after at least 1 week of acclimatization.

In the tea leaf administration experiments, the Yabukita and Benifuki varieties were administered via diet. The composition of the normal feed (Marubeni Nisshin Feed Co., Ltd.) was ≥ 15% crude protein, ≥2% crude fat, ≤5% crude fiber, ≤7% crude ash content, ≥0.4% calcium, ≥0.3% phosphorus, and ≥ 78% total digestible nutrient content. To prevent diarrhea caused by a large amount of green tea leaves in the feed, 2% tea leaves were mixed with the usual feed (10 g of tea leaves in 500 g of normal feed). The mixing of feed with tea leaves was performed just before the intake to minimize oxidation of tea leaf catechin. The tea leaf intake and follow‐up observation periods were 15 and 7 days, respectively. Blood sampling as a control was performed for 5 days before the intake. On the first day of tea leaf intake, sampling of blood was performed at 1, 3, and 5 hr after feeding to avoid anemia, and subsequent blood sampling was performed 2, 3, 4, 5, 8, and 15 days later. The residual quantity in the body was determined in blood samples taken after 1 and 7 days of tea leaf intake. The collected blood was ultrafiltered through an Amicon Ultra centrifugal filter (Merck) and analyzed using HPLC for catechin and caffeine (*n* = 6).

Complete blood counts of white blood cells (WBC), hemoglobin (Hb), and platelets (Plt); renal levels of creatinine (Cre); liver function; and levels of aspartate aminotransferase (AST) and alanine aminotransferase (ALT) were serially assessed.

All the animal experiments were conducted in compliance with the protocol that was reviewed by the Institutional Animal Care and Use Committee and approved by the President of Kagoshima University (Permit Number: #MD14117).

### 2.5 Statistical analysis

The tea infusion and swine intervention experiments were replicated twice independently. Significant differences among all groups were assessed using *t* tests and multiple tests (IBM SPSS Medical Model Statistics 25, Advanced Analytics, Inc.). Data are shown as means ± standard error of the mean (SEM). A probability of *p* < .05 was considered statistically significant.

## 3 RESULTS AND DISCUSSION

### 3.1 Determination of green tea infusion components

**Figure 1:**
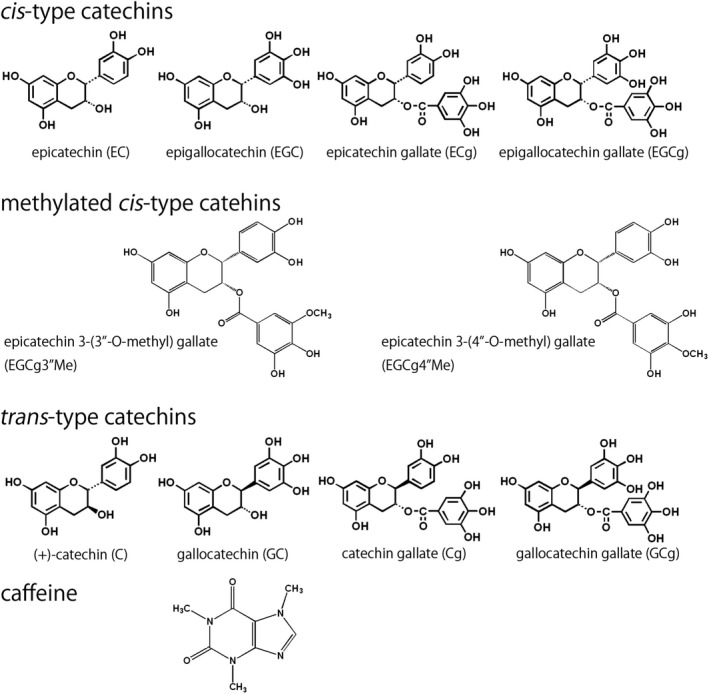
Structures of tea catechins and caffeine

Eight major catechins, two methylated catechins, and caffeine were quantified in the green tea infusion (Figure 1). *Cis*‐type catechins (EC, EGC, ECg, and EGCg) and *trans*‐type catechins ((+)‐C, GC, Cg, and GCg) have different steric configurations for positions 2–3 of the C ring. Methylated catechins are methylated in the gallate group of EGCg. For accurate estimation of tea infusion components, a calibration curve was generated with the standards. The correlation coefficients were all found to be *R* ^2^ > 0.9998, and thus, the concentration could be accurately determined.

Nonrefined and refined tea were eluted at the normal drinking temperatures of 60, 70, and 80°C, and the components contained in the respective infusions are shown in Table 1. EGCg was found to be the most abundant in Benifuki, with GC and caffeine being the second and third most abundant components. EGCg3”Me in Benifuki increased depending on the harvest period. Some studies found the highest concentration of EGCg in green tea infusion (Dalluge, Nelson, Thomas, & Sander, 1998; Khokhar & Magnusdottir, 2002). Comparison between the *cis*‐ and *trans*‐ forms showed that only EGC < GC had the *trans*‐ form in higher levels, whereas the others had the *cis*‐ form in larger quantity ((+)‐C < EC, Cg < ECg, and GCg < EGCg). Moreover, both tea varieties lacked EGCg4”Me, but Benifuki contained EGCg3”Me. Furthermore, as done for Benifuki, eight major catechins and caffeine of Yabukita were quantified in the green tea infusion. The result was very similar to that of Benifuki; however, Yabukita did not contain methylated catechins and contained more caffeine. The results suggested that in both refined Benifuki and Yabukita, the content of each catechin is significantly increased and the caffeine content is significantly reduced compared with those in the nonrefined teas, indicating the importance of the tea refining process.

**Table 1:** Catechins and caffeine content in green tea infusion according to harvest time, manufacturing process, and elution temperature

		EC	EGC	ECg	EGCg	(+)‐C	GC	Cg	GCg	EGCg3”Me	EGCg4”Me	Caffeine
Benifuki (μM)												
Nonrefined tea												
First picking	60°C	8.03 ± 0.02	12.98 ± 0.38	26.85 ± 0.25	65.87 ± 0.28	3.64 ± 0.35^e^	66.54 ± 0.66	n.d.	0.70 ± 0.05	34.03 ± 0.59	n.d	80.31 ± 1.17
	70°C	10.47 ± 0.58^d^	13.78 ± 0.34	35.06 ± 0.74^ad^	80.34 ± 0.99^d^	2.15 ± 0.67^d^	94.71 ± 5.52^d^	n.d	0.93 ± 0.44^a^	47.19 ± 1.00^d^	n.d	102.22 ± 6.06^d^
	80°C	9.82 ± 0.84^d^	47.64 ± 2.58^d^	39.00 ± 1.44^e^	149.67 ± 5.24^e^	3.35 ± 0.43^de^	67.73 ± 4.31	n.d	1.63 ± 0.66^b^	73.89 ± 3.21^e^	n.d	93.13 ± 5.52^d^
Second picking	60°C	7.57 ± 0.29	5.63 ± 0.76^a^	33.43 ± 1.84^a^	50.75 ± 2.98^a^	4.56 ± 0.69	104.83 ± 2.94^a^	n.d	0.38 ± 0.07^a^	26.68 ± 2.05	n.d	79.93 ± 2.39
	70°C	10.99 ± 0.27^d^	13.55 ± 0.23^d^	48.97 ± 1.22^bd^	107.99 ± 2.48^ad^	5.88 ± 0.77^a^	125.88 ± 2.91^ad^	n.d	0.83 ± 0.07^abd^	66.71 ± 2.54^ad^	n.d	109.28 ± 4.79^d^
	80°C	10.58 ± 0.31^d^	46.82 ± 1.41^e^	52.91 ± 1.02^ae^	157.67 ± 3.24^e^	4.37 ± 0.51	74.35 ± 2.71^e^	0.05 ± 0.02^abd^	0.48 ± 0.01^a^	73.00 ± 2.12^e^	n.d	99.94 ± 4.00^d^
Third picking	60°C	11.04 ± 0.78^a^	18.93 ± 0.91^b^	61.69 ± 4.47^bde^	150.05 ± 9.72^b^	6.92 ± 0.31^a^	150.65 ± 7.92^b^	0.10 ± 0.05^a^	0.43 ± 0.16^a^	88.82 ± 7.23^a^	n.d	126.34 ± 7.6^a^
	70°C	10.93 ± 0.43	14.55 ± 0.58	58.48 ± 1.52^d^	132.73 ± 1.72^b^	6.04 ± 0.30^a^	150.49 ± 9.57^b^	0.31 ± 0.26	0.34 ± 0.01^bc^	82.43 ± 2.70^b^	n.d	130.71 ± 3.93^a^
	80°C	11.41 ± 0.62	51.53 ± 3.30^d^	70.04 ± 3.55^be^	231.56 ± 9.39^ad^	6.54 ± 0.46^a^	135.58 ± 8.81^a^	0.84 ± 0.09^cd^	0.41 ± 0.16^a^	126.43 ± 6.74^ad^	n.d	132.74 ± 9.23^a^
Final picking	60°C	10.70 ± 0.34^a^	23.70 ± 0.87^c^	26.62 ± 1.11^de^	83.99 ± 3.73^c^	3.89 ± 0.19	20.75 ± 0.64^c^	n.d	0.22 ± 0.03^a^	118.15 ± 5.00^bde^	n.d	44.63 ± 1.00^b^
	70°C	9.98 ± 0.72	22.50 ± 1.34^a^	21.51 ± 3.14^ce^	68.22 ± 7.81^c^	3.99 ± 0.67^b^	28.16 ± 2.01^cd^	0.18 ± 0.04^d^	n.d	96.19 ± 9.06^ce^	n.d	3.85 ± 3.04^b^
	80°C	10.78 ± 0.70	49.77 ± 2.90^d^	28.10 ± 2.46^cd^	105.62 ± 8.19^bd^	4.03 ± 0.18	0.12 ± 1.77^bd^	0.14 ± 0.04^bd^	0.16 ± 0.04^a^	129.94 ± 12.11^ad^	n.d	45.18 ± 3.32^b^
Refined tea												
First picking	60°C	7.80 ± 0.27	12.98 ± 0.38	29.80 ± 0.44*	78.77 ± 0.96*	4.10 ± 0.84^b^	91.13 ± 1.25*	n.d	0.86 ± 0.42	50.01 ± 0.49*	n.d	91.37 ± 2.57^b^ *
	70°C	11.49 ± 0.49^d^	42.36 ± 7.37^d^ *	42.93 ± 2.12^d^ *	136.48 ± 4.21^d^ *	4.75 ± 0.25*	110.47 ± 9.00^d^	n.d	2.03 ± 0.35*	87.85 ± 4.89^d^	n.d	115.85 ± 6.72^abd^
	80°C	9.70 ± 0.61^e^	46.68 ± 1.20^d^	38.72 ± 2.05^d^	149.65 ± 7.64^e^	3.56 ± 0.36	83.59 ± 2.67*	n.d	1.46 ± 0.87^b^	86.14 ± 8.10^bd^	n.d	104.36 ± 3.52^d^ *
Second picking	60°C	9.80 ± 0.07^a^ *	19.27 ± 0.13^a^ *	47.46 ± 0.75^a^ *	123.36 ± 2.40^a^ *	4.90 ± 0.56^ab^	115.85 ± 1.03^a^ *	0.41 ± 0.06*	1.29 ± 0.08^a^ *	81.76 ± 5.59^a^ *	n.d	99.18 ± 1.72^a^ *
	70°C	11.85 ± 0.65^d^	18.38 ± 0.76^a^ *	56.89 ± 2.91^ad^ *	137.13 ± 6.21^d^ *	7.14 ± 0.33^ad^	137.38 ± 8.91^ad^	0.51 ± 0.05^a^ *	2.26 ± 0.16^d^ *	88.56 ± 6.15*	n.d	121.40 ± 8.69^ad^
	80°C	9.15 ± 0.62*	40.20 ± 1.31^d^ *	45.69 ± 0.99^a^ *	162.12 ± 3.75^ae^	4.24 ± 0.02	85.68 ± 3.64^e^ *	n.d	1.43 ± 0.10^b^ *	88.95 ± 4.90^ab^ *	n.d	91.10 ± 3.77^a^ *
Third picking	60°C	11.89 ± 0.38^b^	25.38 ± 0.59^b^ *	44.47 ± 1.06^b^ *	112.25 ± 2.60^b^ *	6.22 ± 0.06^a^ *	113.25 ± 7.95^a^ *	0.67 ± 0.06*	0.52 ± 0.01^b^	80.36 ± 1.46^a^	n.d	5.72 ± 4.34^bcde^ *
	70°C	11.96 ± 0.56	18.05 ± 0.91^ad^ *	48.28 ± 3.35*	107.07 ± 7.50^a^ *	6.60 ± 0.44^a^	119.79 ± 4.17*	0.42 ± 0.12^acd^	1.97 ± 0.25^d^ *	87.31 ± 7.00	n.d	104.46 ± 3.80^be^ *
	80°C	11.60 ± 0.26^a^	49.01 ± 1.42^e^	45.47 ± 0.75^a^ *	147.30 ± 1.65^d^ *	6.40 ± 0.12^a^	99.07 ± 0.86^ad^ *	0.13 ± 0.02^ae^ *	0.95 ± 0.14^abe^ *	101.47 ± 4.55^ad^ *	n.d	94.31 ± 2.25^ad^ *
Final picking	60°C	6.73 ± 0.26^c^ *	12.26 ± 0.69*	11.33 ± 0.34^c^ *	34.17 ± 1.19^c^ *	1.74 ± 0.55^c^ *	20.29 ± 0.77^b^	0.45 ± 0.17*	n.d	38.91 ± 4.42^b^ *	n.d	34.70 ± 0.91^c^ *
	70°C	8.46 ± 0.24^ad^ *	6.30 ± 0.26^bd^ *	15.45 ± 0.53^bd^ *	32.07 ± 1.31^b^ *	3.70 ± 0.17^bd^	26.92 ± 1.19^bd^	0.27 ± 0.05^bc^	0.20 ± 0.01^ad^ *	40.69 ± 1.38^a^ *	n.d	44.74 ± 1.21^cd^
	80°C	8.52 ± 0.03^d^ *	40.91 ± 0.10^e^ *	16.83 ± 0.09^be^ *	67.60 ± 0.21^be^ *	2.47 ± 0.38^b^ *	25.17 ± 0.03^bd^ *	n.d	n.d	83.56 ± 0.68^bd^ *	n.d	43.32 ± 0.47^bd^
Yabukita (μM)												
Nonrefined tea	60°C	9.8 ± 8.3	23.7 ± 3.4	31.6 ± 2.7	105.0 ± 8.8	5.8 ± 1.2	167.2 ± 1.4	0.9 ± 0.5	0.1 ± 0.0	n.d	n.d	179.8 ± 8.1
	70°C	8.4 ± 5.5	32.1 ± 3.8^d^	40.3 ± 7.8	139.6 ± 5.1^d^	7.9 ± 4.7	201.7 ± 6.7^d^	0.4 ± 0.8	0.3 ± 0.0^d^	n.d	n.d	195.7 ± 9.6
	80°C	8.9 ± 6.1	22.0 ± 1.9	38.0 ± 8.7	118.5 ± 8.8	7.3 ± 4.1	197.1 ± 8.7^d^	0.8 ± 0.4	0.4 ± 0.1^d^	n.d	n.d	183.2 ± 7.3
Refined tea	60°C	10.7 ± 7.4	30.4 ± 9.7	39.8 ± 3.0*	125.6 ± 7.3*	5.2 ± 3.5	97.0 ± 7.2^e^ *	0.4 ± 0.4	1.0 ± 0.9	n.d	n.d	178.0 ± 7.8
	70°C	7.1 ± 1.9	27.1 ± 4.2	28.4 ± 3.7^d^	153.6 ± 3.5^d^ *	3.5 ± 1.3	75.3 ± 7.1^d^ *	0.2 ± 0.2	1.2 ± 0.6	n.d	n.d	178.1 ± 8.7
	80°C	10.0 ± 8.8	49.1 ± 4.3^d^ *	41.5 ± 4.2	153.7 ± 5.2^d^ *	3.9 ± 1.5	87.1 ± 7.3^de^ *	n.d	1.3 ± 1.3	n.d	n.d	199.5 ± 9.3

Note

(+)‐C, catechin; Cg, catechin gallate; EC, epicatechin; ECg, epicatechin gallate; EGC, epigallocatechin; EGCg, epigallocatechin gallate; EGCg3”Me, epigallocatechin‐3‐(3”‐O‐methyl) gallate; EGCg4”Me, epigallocatechin‐3‐(4”‐O‐methyl) gallate; GC, gallocatechin; GCg, gallocatechin gallate; n.d; not detected.

Data are shown as means ± SEM.

^a,b,c^Difference in picking term at same extraction temperature (*p* < .05); ^d,e^difference in temperature at same picking term (*p* < .05).

* *p* < .05 versus nonrefined tea.

Table 1 shows the comparison of tea infusion components according to harvest time, manufacturing process, and elution temperature. ECg, EGCg, GC, GCg, and caffeine were less abundant in final picking tea as compared to those in first, second, and third picking tea. Because catechins are produced in the plant body after receiving sunlight, their content in third picking tea, which receives more sunlight, may be higher than in first picking tea, which is harvested earlier. Therefore, we anticipated that final picking tea would contain higher levels of catechins than other tea samples. Contrary to our expectations, catechins were less abundant in final picking tea compared to those in other tea samples. We speculated that the percentage of total catechins was higher in other tea samples because final picking tea has, instead of soft buds, old leaves and hard stems. Conversely, a comparison between the contents of nonrefined and refined tea showed that ECg and EGCg of the third and final picking tea were higher in nonrefined tea, but the quantities of EC, (+)‐C, and GC in nonrefined and refined tea were similar. Thus, it is clear that the rate of loss of components due to degradation, polymerization, and isomerization is affected by heat‐drying in refining. In addition, EGC, ECg, EGCg, and EGCg3”Me content increased in the infusion solution with increasing elution temperature. In a previous study, high temperature promoted EGCg3”Me extraction efficiency (Maeda‐Yamamoto et al., 2005). Thus, the higher the infusion temperature, the higher the catechin content in the infusion solution.

### 3.2 Determination of tea leaf extract components

In tea leaf extracts, eight major catechins, two methylated catechins, and caffeine were identified and quantified (Table 2). Estimation of catechin and caffeine contents in Benifuki leaf extracts showed that EGCg content is the highest, with EGCg3”Me, caffeine, and GC being the next most abundant components. Comparison of *cis*‐ and *trans*‐ forms in Benifuki showed that only EGC < GC had a higher amount of the *trans*‐ form, whereas the others had the *cis*‐ form in larger quantities ((+)‐C < EC, Cg < ECg, and GCg < EGCg). Furthermore, consistent with the Benifuki infusion, EGCg4”Me was absent from leaves of both varieties, and only EGCg3”Me was detected. Suzuki et al. (2013) also showed that tea leaf extract contains EGCg3”Me, corroborating our results. Caffeine content was significantly reduced in final picking Benifuki.

**Table 2:** Catechins and caffeine content in green tea leaf extracts according to harvest time and manufacturing process

	EC	EGC	ECg	EGCg	(+)‐C	GC	Cg	GCg	EGCg3”Me	EGCg4”Me	Caffeine
Benifuki (μM)											
Nonrefined tea											
First picking	7.98 ± 0.34	32.73 ± 1.41	43.65 ± 1.73	145.59 ± 5.78	3.01 ± 0.20	50.94 ± 2.08	0.15 ± 0.04	2.91 ± 1.27	95.28 ± 3.87	n.d	71.54 ± 3.10
Second picking	10.56 ± 0.44^a^	46.95 ± 2.82^a^	62.96 ± 4.17^a^	200.01 ± 9.74^a^	4.61 ± 0.15^a^	77.51 ± 4.18^a^	0.28 ± 0.01	1.90 ± 0.08^a^	141.57 ± 9.49^a^	n.d	88.93 ± 4.09^a^
Third picking	11.45 ± 0.60^a^	44.72 ± 2.97^a^	77.16 ± 3.32^b^	233.78 ± 9.90^b^	5.81 ± 0.26^b^	99.54 ± 8.98^b^	0.37 ± 0.22	1.34 ± 0.11^a^	164.75 ± 7.31^b^	n.d	104.10 ± 4.44^b^
Final picking	11.51 ± 0.27^a^	46.20 ± 1.07^a^	41.86 ± 0.87	136.97 ± 2.82	4.12 ± 0.28^a^	29.87 ± 8.45^a^	0.67 ± 0.04^a^	0.83 ± 0.06^a^	217.49 ± 3.96^c^	n.d	40.97 ± 0.76^c^
Refined tea											
First picking	10.02 ± 0.51*	43.00 ± 1.42*	60.78 ± 2.26*	212.34 ± 8.00*	2.11 ± 0.20*	72.90 ± 2.34*	0.14 ± 0.02	2.47 ± 0.13	174.42 ± 7.03*	n.d	95.26 ± 0.94*
Second picking	9.82 ± 0.19	36.00 ± 0.75^a^ *	61.90 ± 1.57	190.97 ± 5.26	3.46 ± 0.07^a^ *	80.05 ± 1.48	0.24 ± 0.02^a^ *	2.64 ± 0.08*	120.65 ± 3.18^a^ *	n.d	81.79 ± 1.87
Third picking	13.08 ± 0.19^a^ *	45.41 ± 0.66	66.10 ± 0.55^a^ *	189.58 ± 2.97^a^ *	5.33 ± 0.52	98.48 ± 1.38	0.33 ± 0.02	2.61 ± 0.09*	192.10 ± 2.97^b^ *	n.d	90.53 ± 1.84^b^ *
Final picking	8.56 ± 0.25^b^ *	28.39 ± 0.86^b^ *	28.96 ± 0.87^b^ *	105.43 ± 2.96^b^ *	2.86 ± 0.15^a^ *	28.67 ± 0.87	0.62 ± 0.02	0.41 ± 0.02^a^ *	186.68 ± 5.42^b^ *	n.d	40.73 ± 1.08
Yabukita (μM)											
Nonrefined tea	15.3 ± 3.2	52.2 ± 9.5	53.9 ± 2.5	153.2 ± 5.3	9.5 ± 1.4	299.6 ± 6.1	1.5 ± 0.0	0.6 ± 0.1	n.d	n.d	217.5 ± 7.5
Refined tea	16.3 ± 5.3	53.2 ± 1.5	56.7 ± 7.4	192.4 ± 12.8*	9.2 ± 1.7	235.1 ± 4.4*	1.2 ± 0.6	1.9 ± 0.2*	n.d	n.d	288.5 ± 2.7*

Note

Data are shown as means ± SEM.

Abbreviations: (+)‐C, catechin; Cg, catechin gallate; EC, epicatechin; ECg, epicatechin gallate; EGC, epigallocatechin; EGCg, epigallocatechin gallate; EGCg3”Me, epigallocatechin‐3‐(3”‐O‐methyl) gallate; EGCg4”Me, epigallocatechin‐3‐(4”‐O‐methyl) gallate; GC, gallocatechin; GCg, gallocatechin gallate; n.d; not detected.

*p* < .05 versus first picking.

*p* < 0.05 versus second picking.

*p* < 0.05 versus third picking.

*p* < 0.05 versus final picking.

* *p* < 0.05 versus nonrefined tea.

Table 2 shows the comparison of tea leaf extract components according to harvest time and manufacturing process. The ECg, EGCg, GC, GCg, and caffeine contents in Benifuki were lower in final picking tea than those in first, second, and third picking tea. Similar to the tea infusion results, few differences were found in the contents of nonrefined and refined tea extracts. GC was more abundant in the tea infusion than in tea leaf extracts because other catechins change to GC by heating of green tea leaves, whereas ECg, EGCg, Cg, GCg, and EGCg3”Me were more abundant in Benifuki leaf extracts than in the tea infusion. Furthermore, eight major catechins and caffeine were detected and quantified in Yabukita leaf extracts, as had been done for Benifuki. The result was similar to that of Benifuki; however, Yabukita did not contain methylated catechins and contained more caffeine.

### 3.3 Absorption of catechins in miniature swine

**Figure 2:**
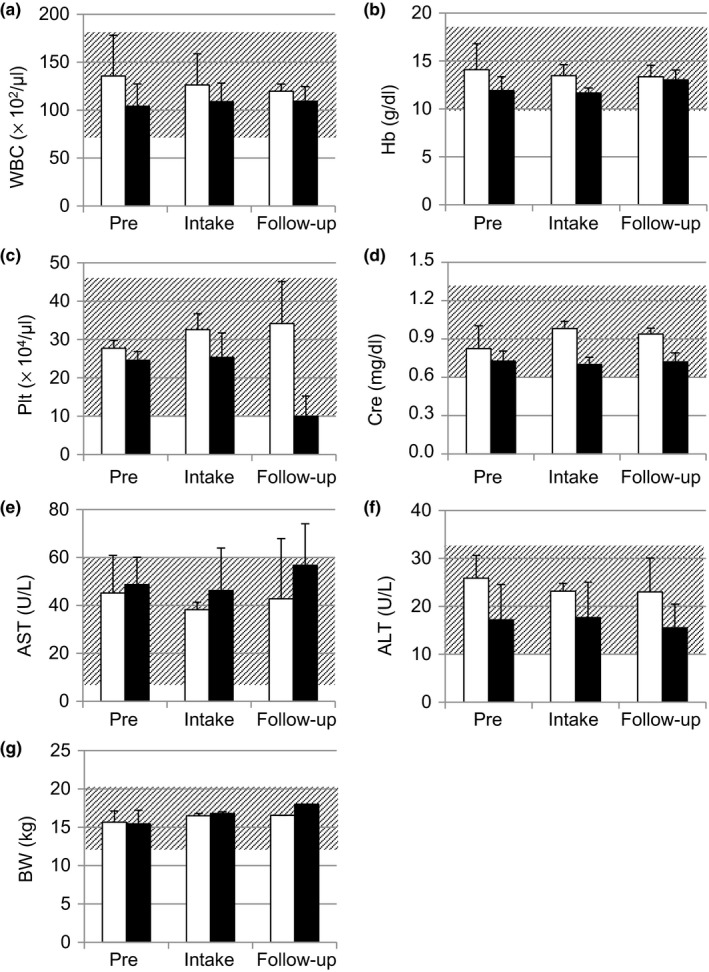
Basic blood component levels in miniature swine fed green tea leaves. (a) WBC, white blood cell counts; (b) Hb, hemoglobin; (c) Plt, platelets; (d) Cre, creatinine; (e) AST, aspartate aminotransferase; (f) ALT, alanine aminotransferase; g: BW, body weight. pre, average values determined as controls for 5 days before tea leaf intake; intake, average values during the period (15 days) of tea leaf intake; follow‐up, average values during the follow‐up period (7 days) after ingestion of tea leaves. White bar: Yabukita; Black bar: Benifuki. Gray shading represents the range of mean values corresponding to normal 4‐month‐old CLAWN miniature swine. * *p* < .05 versus Yabukita

Because miniature swine are omnivorous, the addition of green tea leaves did not change their feed acceptability. When the intake quantity of tea leaves was over 5%, loose stool was observed (data not shown). In the present experiments, therefore, the quantity of tea leaves in the feed was kept at 2% to maintain normal stool condition; consequently, abnormal stool excretion was not observed. In addition, Figure 2 shows that the animals had normal levels of WBC, Hb, Plt, Cre, AST, ALT, and BW during the experimental period. The mean values ± standard error (SE) of blood tests of normal 4‐month‐old CLAWN miniature swine were as follows: WBC, 129 ± 38 × 10^2^/μl; Hb, 14.2 ± 0.7 g/dl; Plt, 32.4 ± 7.2 × 10^4^/μl; Cre, 1.0 ± 0.7 mg/dl; AST, 19.3 ± 5.3 U/L; and ALT, 37.2 ± 7.6 U/L. Miniature swine used in this study presented normal levels of WBC, Hb, Plt, Cre, and BW. AST and ALT levels were within the acceptable error range of individual difference. These normal levels were constant through the study period (pre, intake, and follow‐up). Plt in the follow‐up period was at the lower limit of normal levels (Figure 2c), but the reason was unknown. No change in BW was observed during the experimental period (Figure 2g).

The amount of catechins and caffeine from Yabukita and Benifuki tea varieties contained in miniature swine blood serum is shown in Table 3. Green tea leaves are abundant in ECg and EGCg, but these compounds were rarely detected in the serum of the animals. Presumably, the galloyl moiety (gallate) was not detected because it is hydrolyzed during digestion. EGC, EGCg, (+)‐C, GC, and caffeine were significantly absorbed at 1 hr after feeding. EC, ECg, Cg, and GCg from both Yabukita and Benifuki varieties were not significantly absorbed by miniature swine (<1 μM). (+)‐C was absorbed mostly on day 5 of Benifuki tea intake but was not detected after day 2 of Yabukita tea intake. EGC was absorbed mostly on days 5 and 15 of Benifuki and Yabukita tea intake, respectively. Renouf et al. (2011) quantified the green tea catechins in plasma and revealed that EGC was the most bioavailable catechin. The absorbance pattern of GC was very similar between Yabukita and Benifuki varieties. EGCg4”Me was detected at significantly high levels during the entire intake period, but EGCg3”Me was not detected in the blood of miniature swine fed Benifuki tea. It is possible that the position of the methyl group was modified by the pig metabolism. Furthermore, caffeine from both Yabukita and Benifuki tea varieties was found to be easily accumulated in miniature swine. Kajiya, Kumazawa, and Nakayama (2001) investigated the interaction of tea catechins with lipid bilayers using liposome systems and demonstrated that different stereochemical structures influence the affinity for lipid bilayers and biological activities.

**Table 3:** Catechins and caffeine content in the serum of miniature swine fed Benifuki and Yabukita tea varieties

Sampling	EC	EGC	ECg	EGCg	(+)‐C	GC	Cg	GCg	EGCg3”Me	EGCg4”Me	Caffeine
Benifuki (μM)											
pre	n.d	n.d	n.d	n.d	n.d	n.d	n.d	n.d	n.d	n.d	n.d
1h	n.d	0.22 ± 0.00*	n.d	1.12 ± 0.42*	0.20 ± 0.03*	115.94 ± 49.26*	n.d	n.d	n.d	7.56 ± 1.32*	3.50 ± 1.23*
3h	n.d	0.50 ± 0.22*	n.d	0.62 ± 0.29*	0.26 ± 0.16*	109.49 ± 5.61*	n.d	n.d	n.d	3.31 ± 0.21*	6.39 ± 2.19*
5h	n.d	1.39 ± 0.49*	n.d	n.d	0.11 ± 0.08	48.37 ± 27.48*	0.08 ± 0.02*	n.d	n.d	5.19 ± 1.94*	9.47 ± 1.73*
d2	n.d	1.47 ± 0.90*	n.d	n.d	1.03 ± 0.24*	84.13 ± 24.61*	0.08 ± 0.01*	n.d	n.d	0.56 ± 0.09*	10.18 ± 4.87*
d3	n.d	7.21 ± 2.07*	n.d	n.d	n.d	91.71 ± 24.01*	0.10 ± 0.01*	n.d	n.d	7.79 ± 2.70*	6.66 ± 1.73*
d4	n.d	10.92 ± 2.52*	n.d	n.d	0.36 ± 0.20*	107.04 ± 21.26*	n.d	n.d	n.d	2.52 ± 0.25*	15.28 ± 3.37*
d5	n.d	103.67 ± 44.50*	n.d	n.d	11.14 ± 2.79*	84.31 ± 31.81*	n.d	n.d	n.d	2.56 ± 0.54*	21.98 ± 1.18*
d8	n.d	52.96 ± 5.89*	n.d	6.94 ± 0.34*	4.49 ± 0.01*	110.29 ± 34.69*	n.d	n.d	n.d	1.44 ± 0.43*	7.41 ± 2.13*
d15	n.d	11.50 ± 2.17*	n.d	0.35 ± 0.09*	0.16 ± 0.29	164.42 ± 43.43*	0.30 ± 0.12*	n.d	n.d	2.86 ± 1.10*	7.29 ± 7.02
d16	n.d	9.90 ± 5.71*	n.d	9.56 ± 7.71*	n.d	129.47 ± 41.86*	0.14 ± 0.06*	0.60 ± 0.51	n.d	1.49 ± 0.98	7.65 ± 7.81
d22	n.d	0.16 ± 0.01*	n.d	n.d	n.d	68.76 ± 12.74*	0.07 ± 0.01*	0.41 ± 0.04*	n.d	0.11 ± 0.01*	0.03 ± 0.02
Yabukita (μM)											
pre	n.d	n.d	n.d	n.d	n.d	n.d	n.d	n.d	n.d	n.d	n.d
1h	n.d	1.19 ± 0.37*	n.d	0.34 ± 0.03*	0.21 ± 0.32	84.99 ± 10.07*	n.d	n.d	n.d	n.d	2.19 ± 1.52
3h	n.d	1.80 ± 1.70	n.d	0.75 ± 0.25*	0.14 ± 0.05*	143.60 ± 32.82*	n.d	n.d	n.d	n.d	6.17 ± 3.05*
5h	n.d	12.27 ± 5.75*	n.d	0.81 ± 0.48*	0.33 ± 0.23	128.24 ± 35.70*	n.d	n.d	n.d	n.d	3.88 ± 1.37*
d2	n.d	5.91 ± 4.91	n.d	n.d	9.99 ± 6.84	99.26 ± 35.49*	n.d	n.d	n.d	n.d	10.44 ± 0.42*
d3	n.d	24.46 ± 7.15*	n.d	n.d	n.d	95.73 ± 22.34*	n.d	n.d	n.d	n.d	29.52 ± 11.98*
d4	n.d	54.45 ± 43.16	0.62 ± 0.22*	n.d	n.d	74.87 ± 52.95	0.41 ± 0.04*	n.d	n.d	n.d	35.91 ± 28.50
d5	n.d	36.81 ± 24.80	0.71 ± 0.07*	n.d	n.d	98.53 ± 51.71*	0.65 ± 0.06*	n.d	n.d	n.d	34.66 ± 21.39*
d8	n.d	31.10 ± 13.52*	0.47 ± 0.05*	n.d	n.d	130.74 ± 31.73*	0.50 ± 0.25*	n.d	n.d	n.d	57.17 ± 24.16*
d15	n.d	43.77 ± 17.39*	n.d	n.d	n.d	177.61 ± 37.32*	0.42 ± 0.13*	n.d	n.d	n.d	19.06 ± 3.57*
d16	n.d	24.98 ± 6.02*	n.d	n.d	n.d	162.01 ± 40.00*	0.43 ± 0.07*	n.d	n.d	n.d	20.64 ± 5.00*
d22	n.d	2.41 ± 0.18*	n.d	n.d	n.d	111.38 ± 25.50*	n.d	n.d	n.d	n.d	1.39 ± 0.62*

Note

Sampling of blood was performed at 1, 3, and 5 hr after feeding to avoid anemia, and subsequent blood sampling was performed 2, 3, 4, 5, 8, and 15 days after. Data are shown as means ± SEM.

Abbreviations: (+)‐C, catechin; Cg, catechin gallate; EC, epicatechin; ECg, epicatechin gallate; EGC, epigallocatechin; EGCg, epigallocatechin gallate; EGCg3”Me, epigallocatechin‐3‐(3”‐O‐methyl) gallate; EGCg4”Me, epigallocatechin‐3‐(4”‐O‐methyl) gallate; GC, gallocatechin; GCg, gallocatechin gallate; n.d; not detected.

* *p* < 0.05 versus pre.

## 4 CONCLUSION

We studied the catechin composition of the general green tea varieties Yabukita and Benifuki, including methylated catechins. In the present study, we found that the catechin and caffeine contents vary according to the harvest period and infusion temperature in the Benifuki variety. Interestingly, catechin contents were influenced by harvest time; some catechins were more easily extracted with increased temperature. Accordingly, the taste of the tea infusion may be influenced by the amount of catechins. For example, the concentration of EGC in the nonrefined first picking Benifuki tea infusion was the highest with 80°C elution. The differences between the tea leaf extracts of Benifuki and Yabukita are as follows: (a) Yabukita contains a higher content of GC generated by isomerization; (b) Yabukita does not contain methylated catechins; and (c) the caffeine content of Yabukita is high. Thus, leaf extracts of nonrefined final picking Benifuki tea, which contains a high amount of EGCg3”Me and low quantity of caffeine, are considered optimal for consumption by children and pregnant women. In order to investigate the absorption of catechins and caffeine contained in tea leaves, it is important to evaluate in vivo metabolism using models with digestive and vascular functions similar to those of humans, such as miniature swine. In this metabolic study, although the tea leaves contained EC, it was not detected in the blood of miniature swine fed the same. ECg from Yabukita was detected only on days 4–8 because a hydroxyl group of ring B of EGCg was removed during metabolism. The transient detection of Cg and GCg from Benifuki was due to the isomerization of EGCg during metabolism. On the other hand, EGC and GC were detected at significantly high levels after 1 hr in animals fed both varieties. EGCg3”Me was detected in tea infusion and leaf extracts, whereas EGCg4”Me was detected in the blood. This may result from a change in the position of the methyl group during metabolism, although only a small quantity of EGCg4”Me was detected in the blood. Of the green tea varieties, Benifuki has a peculiar combination of odor and bitterness, and thus, some consumers prefer other varieties. The results of the present study suggest that nonrefined September–October final picking tea (autumn and winter tea) of the Benifuki variety is preferable over the Yabukita variety for consumption by children and pregnant women, which could lead to increased consumption of the Benifuki variety.

## CONFLICT OF INTEREST

The authors declare that they do not have any conflict of interest.

## ETHICAL REVIEW

All the animal experiments were conducted in compliance with the protocol that was reviewed by the Institutional Animal Care and Use Committee and approved by the President of Kagoshima University (Permit Number: #MD14117).

## References

[fsn31143-bib-0001] Amarowicz, R. , Pegg, R. B. , & Bautista, D. A. (2000). Antibacterial activity of green tea polyphenols against *Escherichia coli* K 12. Nahrung/Food, 44(1), 60–62. 10.1002/(SICI)1521-3803(20000101)44:1<60:AID-FOOD60>3.0.CO;2-L 10703004

[fsn31143-bib-0002] Dalluge, J. J. , Nelson, A. C. , Thomas, J. B. , & Sander, L. C. (1998). Selection of column and gradient elution system for the separation of catechins in green tea using high‐performance liquid chromatography. Journal of Chromatography A, 793(2), 265–274. 10.1016/S0021-9673(97)00906-0 9474785

[fsn31143-bib-0003] Dascombe, M. J. , & Milton, A. S. (1972). The effect of caffeine on the antipyretic action of aspirin administered during endotoxin induced fever. British Journal of Pharmacology, 46(3), 548–549. PMC1666520 4656630

[fsn31143-bib-0004] Fukai, K. , Ishigami, T. , & Hara, Y. (1991). Antibacterial activity of tea polyphenols against phytopathogenic bacteria. Agricultural and Biological Chemistry, 55(7), 1895–1897. 10.1271/bbb1961.55.1895

[fsn31143-bib-0005] Jain, N. , Siddiqi, M. , & Weisburger, J. (2006). Protective effects of tea on human health. Wallingford, Oxfordshire, UK: CABI International.

[fsn31143-bib-0006] Kajiya, K. , Hojo, H. , Suzuki, M. , Nanjo, F. , Kumazawa, S. , & Nakayam, T. (2004). Relationship between antibacterial activity of (+)‐catechin derivatives and their interaction with model membrane. Journal of Agricultural and Food Chemistry, 52(6), 1514–1519. 10.1021/jf0350111 15030204

[fsn31143-bib-0007] Kajiya, K. , Kumazawa, S. , & Nakayama, T. (2001). Steric effects on interaction of tea catechins with lipid bilayers. Bioscience, Biotechnology, and Biochemistry, 65(12), 2638–2643. 10.1271/bbb.65.2638 11826958

[fsn31143-bib-0008] Khokhar, S. , & Magnusdottir, S. G. M. (2002). Total phenol, catechin, and caffeine contents of teas commonly consumed in the United Kingdom. Journal of Agricultural and Food Chemistry, 50(3), 565–570. 10.1021/jf010153l 11804530

[fsn31143-bib-0009] Kuroda, Y. , & Hara, Y. (2004). Health effects of tea and its catechins. New York, NY: Springer.

[fsn31143-bib-0010] LaJambe, C. M. , Kamimori, G. H. , Belenky, G. , & Balkin, T. J. (2005). Caffeine effects on recovery sleep following 27 h total sleep deprivation. Aviation, Space, and Environmental Medicine, 76(2), 108–113. 15742825

[fsn31143-bib-0011] Lambert, J. D. , & Yang, C. S. (2003). Cancer chemopreventive activity and bioavailability of tea and tea polyphenols. Mutation Research, 523, 201–208. 10.1016/S0027-5107(02)00336-6 12628518

[fsn31143-bib-0012] Maeda‐Yamamoto, M. , Ema, K. , Monobe, M. , Shibuichi, I. , Shinoda, I. , Yamamoto, T. , & Fujisawa, T. (2009). The efficacy of early treatment of seasonal allergic rhinitis with benifuuki green tea containing O‐methylated catechin before pollen exposure: An open randomized study. Allergology International, 58, 437–444. 10.2332/allergolint.08-OA-0066 19542766

[fsn31143-bib-0013] Maeda‐Yamamoto, M. , Nagai, H. , Suzuki, Y. , Ema, K. , Kanda, E. , & Mitsuda, H. (2005). Changes in O‐methylated catechin and chemical component contents of ‘Benifuuki’ green tea (*Camellia sinensis L.*) beverage under various extraction conditions. Food Science and Technology Research, 11(3), 248–253. 10.3136/fstr.11.248

[fsn31143-bib-0014] Muramatsu, K. , Fukuyo, M. , & Hara, Y. (1986). Effect of green tea catechins on plasma cholesterol level in cholesterol‐fed rats. Journal of Nutritional Science and Vitaminology, 32(6), 613–622. 10.3177/jnsv.32.613 3585557

[fsn31143-bib-0015] Murase, T. , Nagasawa, A. , Suzuki, J. , Hase, T. , & Tokimitsu, I. (2002). Beneficial effects of tea catechins on diet‐induced obesity: Stimulation of lipid catabolism in the liver. International Journal of Obesity and Related Metabolic Disorders, 26(11), 1459–1464. 10.1038/sj.ijo.0802141 12439647

[fsn31143-bib-0016] Renouf, M. , Redeuil, K. , Longet, K. , Marmet, C. , Dionisi, F. , Kussmann, M. , … Nagy, K. (2011). Plasma pharmacokinetics of catechin metabolite 4’‐O‐Me‐EGC in healthy humans. European Journal of Nutrition, 50(7), 575–580. 10.1007/s00394-010-0164-1 21212969

[fsn31143-bib-0017] Rodríguez‐Artalejo, F. , & Lopez‐Garcia, E. (2017). Coffee consumption and cardiovascular disease: A condensed review of epidemiological evidence and mechanisms. Journal of Agricultural and Food Chemistry, 66(21), 5257–5263. 10.1021/acs.jafc.7b04506 29276945

[fsn31143-bib-0018] Stookey, J. D. (1999). The diuretic effects of alcohol and caffeine and total water intake misclassification. European Journal of Epidemiology, 15(2), 181–188. 10204649 10.1023/a:1007559725607

[fsn31143-bib-0019] Suzuki, T. , Kumazoe, M. , Kim, Y. , Yamashita, S. , Nakahara, K. , Tsukamoto, S. , … Tachibana, H. (2013). Green tea extract containing a highly absorbent catechin prevents diet‐Induced lipid metabolism disorder. Scientific Reports, 3, 2749 10.1038/srep02749 24067358 PMC3782887

[fsn31143-bib-0020] Yokozawa, T. , Cho, E. J. , Hara, Y. , & Kitani, K. (2000). Antioxidative activity of green tea treated with radical initiator 2,2'‐azobis(2‐amidinopropane) dihydrochloride. Journal of Agricultural and Food Chemistry, 48(10), 5068–5073. 10.1021/jf000253b 11052779

[fsn31143-bib-0021] Yokozawa, T. , Okura, H. , Sakanaka, S. , Ishigaki, S. , & Kim, M. (1994). Depressor effect of tannin in green tea on rats with renal hypertension. Bioscience, Biotechnology, and Biochemistry, 58, 855–858. 10.1271/bbb.58.855

